# Porous Biocarbon Derived from Garlic Peel Biowaste for Symmetric Supercapacitor Applications: Electrochemical Performance in Aqueous and Organic Electrolytes

**DOI:** 10.1021/acsomega.6c03559

**Published:** 2026-09-21

**Authors:** Santhosh Ravichandran, Manuel Octavio Fuentez-Torres, Ivonne Liliana Alonso-Lemus, Beatriz Escobar-Morales, Francisco Javier Rodríguez-Varela

**Affiliations:** † Nanociencias y Nanotecnología, Cinvestav Saltillo, Av. Industria Metalúrgica 1062, Ramos Arizpe, Coahuila C.P. 25900, Mexico; ‡ Secihti-Cinvestav Saltillo, Sustentabilidad de los Recursos Naturales y Energía, Av. Industria Metalúrgica 1062, Parque Industrial Ramos Arizpe, Ramos Arizpe, Coahuila C.P. 25900, Mexico; § Secihti, Centro de Investigación Científica de Yucatán, Unidad de Energía Renovable, Calle 43, No. 130 Col. Chuburná de Hidalgo, Mérida, Yucatán C.P. 97200, Mexico; ∥ Sustentabilidad de los Recursos Naturales y Energía, Cinvestav Saltillo, Av. Industria Metalúrgica 1062, Ramos Arizpe, Coahuila C.P. 25900, Mexico

## Abstract

Biomass is an inexpensive source of carbon, from which added-value energy materials can be produced. On this matter, this research utilizes a hydrothermal process with pyrolysis treatment to produce a Garlic Peel Biocarbon (GPB) using the outer layers of the species as the source of carbon. The as-produced GPB shows a high specific surface area (SSA) of 1240.7 m^2^ g^–1^ and a pore diameter of 3.96 nm, indicating its hierarchical mesoporous structure. GPB has been characterized as active material in a symmetrical GPB||GPB supercapacitor, displaying high cycle stability in 6 M KOH, 0.5 M Na_2_SO_4_, and 1 M NaClO_4_–(EC|PC) (ethylene carbonate|propylene carbonate). Even though the specific capacity (*C*_sp_) in KOH is the highest at 1 mV s^–1^ (112.5 F g^–1^), those with Na_2_SO_4_ and the organic electrolyte are not significantly lower (103.2 and 99.2 F g^–1^, respectively). More importantly, the supercapacitor achieves an operating voltage window of 1.0, 1.8, and 2.5 V in KOH, Na_2_SO_4_, and NaClO_4_, respectively. Hence, there is an enhanced behavior in the organic electrolyte that has promoted the device to attain energy density (*E*_D_) and power density (*P*_D_) values of 19.9 Wh kg^–1^ and 183.9 W kg^–1^, respectively. The results demonstrate that active material-electrolyte conformation strongly influences the balance between *C*_sp_, operating voltage window, *E*_D_, and *P*_D_. Here, the GPB–NaClO_4_ system enables a wider voltage window, outperforming the other configurations in terms of *E*_D_ and *P*_D_, as shown in the Ragone plot and radar chart. The findings indicate that GPB has excellent performance and significant promise as an active electrode material for supercapacitors.

## Graphical abstract

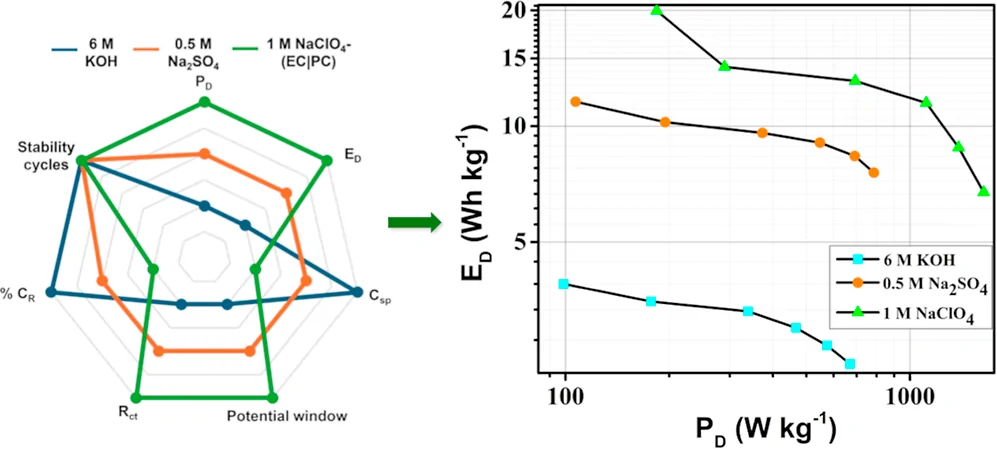

## 1 Introduction

Supercapacitors are pivotal technologies in electrochemical energy storage due to their high power density, rapid charge–discharge kinetics, and long-term cyclability.^1,2^ They exhibit exceptional durability, in some cases retaining over 90% of their initial capacitance after 100,000 charge–discharge cycles.^3,4^ This performance enables their integration into systems requiring frequent energy bursts, such as regenerative braking in electric vehicles, grid frequency stabilization, and peak power management in renewable energy systems, underscoring their versatility across energy-intensive applications.^5,6^ Although advanced batteries possess a higher energy density in comparison, ongoing investigations are concentrated on augmenting the energy storage capability of supercapacitors via the improvement of performance by developing novel electrodes.^7,8^

Some characteristics of electrode active materials for supercapacitors, such as SSA, pore size distribution, and electrical conductivity, have a substantial impact on the performance of these devices.^8^ Porous carbon-based materials with high SSA enable good ion accumulation through an increased number of active sites, leading to improved *C*_sp_.^9,10^ Additionally, an adequate pore size promotes ion transport and accessibility of ions, while electrical conductivity governs the speed of charge transfer in the electrode material.^11,12^

Considering their naturally acquired advantageous features, such as self-doping with heteroatoms, complex chemical composition that may include activity-promoting metal sites, ease of being obtained with high SSA values, large biomass availability, and their intrinsic eco-friendly attributes, biocarbons have attracted considerable attention as an alternative to conventional nanostructured carbons.^13^ More so, since the environmental impact and cost of nanostructured carbons are substantially high.^14^ Thus, in recent years, biocarbons have emerged as a prominent alternative for supercapacitor applications.^15,16^

A variety of biomass precursors, such as coconut shells, wood, and agricultural waste, have been explored for the production of biocarbons aimed at supercapacitors.^17,18^ Typically, biomass undergoes transformation into porous carbon structures via hydrothermal treatment, followed by activation by pyrolysis, which causes structural modifications, including the development of graphitic or carbon domains, enhancing the degree of graphitization and electrical conductivity of the biocarbon, hence directly improving charge transfer and performance in supercapacitor electrodes. Even more, heat treatment at relatively high temperatures by pyrolysis creates pores and increases the SSA of the resulting biocarbon.^19,20^

Moreover, biocarbons often retain heteroatoms such as nitrogen (N) and sulfur (S) from their organic biowaste precursor, which are known to enhance surface reactivity and capacitive behavior, among other electrochemical properties that enhance the *E*_D_ and *P*_D_ achieved by supercapacitors.^21,22^

On this issue, garlic is a widely cultivated species around the world, with a total production of 2.9 million metric tons recorded in 2020.^23,24^ China stands as the leading producer, accounting for 73% of global output. Other notable countries that produce garlic are India, Bangladesh, and Egypt.^22^ Furthermore, garlic peel (GP), a lignocellulosic residue, is particularly notable for its intrinsic heteroatom content and ion-transport capabilities associated with its vascular structure during metabolic processes.^25,26^ These inherent features suggest that GP has the potential to form hierarchical porous networks after heat treatment.

GP biocarbons (GPBs) have been evaluated in supercapacitor applications. For instance, an *E*_D_ of 14.65 Wh kg^–1^ at a *P*_D_ of 310.67 W kg^–1^ has been reported from the GHC-17 3D hierarchical porous carbon, obtained from carbonization and pyrolysis processes of garlic skin in an asymmetric supercapacitor operating with 6 M KOH electrolyte.^27^ Also, the so-called dual-frequency ultrasonic-assisted enzymolysis strategy, followed by carbonization-activation, has been implemented to produce a GP porous carbon.^28^ In a symmetric supercapacitor with 6 M KOH equipped with DUGPC electrodes, the GP porous material achieved an *E*_D_ of 15.78 Wh kg^–1^ at a *P*_D_ of 50.04 W kg^–1^.

Moreover, an activated carbon obtained from GP generated *E*_D_ = 28.58 Wh kg^–1^ with *P*_D_ = 71.16 W kg^–1^ in 1 M H_2_SO_4_ electrolyte.^29^ Recently, a functionalized nanoporous biocarbon has been produced from garlic waste after an activation process by pyrolysis.^30^ In a symmetric supercapacitor with 3 M KOH, the GAAC6 nanoporous biocarbon achieved an *E*_D_ of 31.8 Wh kg^–1^ at a high *P*_D_ of 8146 W kg^–1^. Therefore, GPB shows relevant properties for supercapacitor applications.

In addition, the choice of electrolyte has a considerable impact on the performance of supercapacitors. Economically viable aqueous electrolytes such as KOH offer high ionic conductivity. Nonetheless, KOH faces limitations when implemented in supercapacitors due to a narrow operating voltage window,^31^ which may be limited to ca. 1.0–1.2 V.^25,28^ Normally, organic electrolytes such as NaClO_4_ and TEABF_4_, either in acetonitrile (ACN) or propylene carbonate|ethylene carbonate (PC|EC), offer a wider electrochemical stability window in supercapacitors, which leads to higher *E*_D_ output.

Electrochemical stability windows in the 2.1–2.6 V range have been reported for NaClO_4_.^32,33^ However, one important drawback of NaClO_4_ is its lower ionic conductivity compared to that of KOH.^34,35^ Therefore, the quest for electrolytes that provide higher *E*_D_ and *P*_D_ values from specific electrodes in supercapacitors is today an open area of research. Even more, considering the increasing interest in studies related to the development of novel biocarbon active materials.

In this study, a greener and less chemically aggressive procedure to obtain GPB is proposed, compared to previous works.^25,27–30^ In a first step, the biomass is submitted to hydrothermal treatment in acetic acid, avoiding the carbonization process. Then, pyrolysis is carried out with ZnCl_2_ as an activator, which interacts differently with the biomass and is less aggressive than the more widely used KOH strong base.

Moreover, in contrast with existing research based on garlic-based active materials for supercapacitors in which one single electrolyte has been evaluated,^25,27–30^ in this work, three electrolytes, i.e., alkaline aqueous (6 M KOH), neutral aqueous (0.5 M Na_2_SO_4_), and organic (1 M NaClO_4_–(EC/PC)), have been used in the device. The aim is to elucidate their specific contribution based on characteristics like ionic conductivity and voltage stability and then find a correlation between electrolyte properties and GPB electrochemical behavior.

Such a configuration allows us to evaluate the performance of GPB in the different environments and thus determine the effect of the active material–electrolyte system on symmetric supercapacitor output values. The performance comparison in the three selected environments is summarized in terms of the Ragone plot and radar chart results, which may help in the decision-making to select the adequate electrolyte for explicit supercapacitor applications with GPB as an active material.

## 2 Experimental Section

### 2.1 Materials

GP (*Allium sativum* L.) was obtained from the National Institute of Forestry, Agriculture, and Livestock (INIFAP) in Aguascalientes, Mexico, registered as the “*San Marqueño*” variety under certification code AJO-009-231110. Analytical-grade chemical reagents were employed without additional purification steps: 2-propanol (C_3_H_8_O, 99.5%), nitric acid (HNO_3_, 70%), ethylene carbonate (C_3_H_4_O_3_, 98%), acetic acid (C_2_H_4_O_2_, ≥99.7%), zinc chloride (ZnCl_2_, ≥98%), potassium hydroxide (KOH, 90%), propylene carbonate (C_4_H_6_O_3_, 99.7%), sodium sulfate (Na_2_SO_4_, ≥99%), sodium perchlorate (NaClO_4_, ≥98%), stainless steel mesh, and Whatman chromatography paper (glass fiber) were procured from Sigma-Aldrich. Polyvinylidene fluoride (−CH_2_CF_2_−) was purchased from Alfa Aesar, while Vulcan XC-72 was supplied by Cabot Corp. Hydrochloric acid (HCl, 37.3%) was acquired from Fermont. Ultrapure nitrogen (N_2_) was obtained from Infra.

### 2.2 Synthesis of Garlic Peel Biocarbon (GPB)

GP underwent hydrothermal carbonization under the following conditions: 1 g of dried material was dispersed in 20 mL of acetic acid (0.23 M solution) and loaded into an autoclave reactor, where it was treated at 200 °C for 12 h. The resulting hydrocarbon (GPH) was dehydrated in a furnace at 60 °C for 12 h. To chemically activate the GPH, ZnCl_2_ was employed in a carbon-to-activator 1:2 mass ratio. The mixture was homogenized via agitation at 80 °C for 4 h, followed by drying at 110 °C for 18 h to form a consistent precursor paste. This paste was subjected to pyrolysis in N_2_ atmosphere at 800 °C for 2 h, with a controlled heating rate of 10 °C per min. Postpyrolysis, the product was purified through sequential washing with 1 M HCl and deionized water, then dried at 60 °C for 12 h, yielding the final activated biocarbon designated as GPB. The yield achieved for GPB was 14.55%.

### 2.3 Physicochemical Characterization of GPB

Elemental composition analysis and spatial mapping were conducted utilizing a Philips XL30 Scanning Electron Microscope (SEM) integrated with an energy-dispersive X-ray spectroscopy (EDS) detector, operated at an accelerating voltage of 20 kV. Morphological characterization was performed via a JEOL JSM-7800F Prime Field Emission SEM (FE-SEM), providing high-resolution morphological imaging. Crystalline phase identification was achieved through X-ray diffraction (XRD) using a Bruker D2 PHASER diffractometer with Cu Kα radiation (λ = 0.154184 nm), scanning a 2θ angular range of 10–100°. Raman spectra were acquired with a Thermo Scientific DXR2Microscope employing a He–Ne laser (λ = 630 nm), covering wavenumbers from 200 to 4000 cm^–1^.

Fourier Transform Infrared (FT-IR) spectroscopy analysis was executed with a Bruker Tensor II spectrometer, capturing vibrational modes across the 4000–400 cm^–1^ interval. Textural properties were determined via N_2_ adsorption–desorption isotherms using a Quantachrome NovaWin analyzer, with Brunauer–Emmett–Teller (BET) methodology applied for surface area evaluation. X-ray photoelectron spectroscopy (XPS) measurements were recorded using a Thermo Scientific K-Alpha spectrometer to probe surface chemistry. Spectral deconvolution and compositional analysis were conducted using CasaXPS software, ensuring precise peak fitting and elemental state resolution.

### 2.4 Electrochemical Characterization

Experiments were conducted in a Biologic bipotentiostat (VSP-300) by utilizing a conventional three-electrode setup. An Ag/AgCl in saturated KCl solution was used as the reference electrode, while the counter electrode was a graphite bar.

To manufacture the working electrodes, a weight ratio of 10:80:10 of carbon black, active material, and binder (PVDF) was used. A tiny amount of propanol was blended into the mixture and homogenized to achieve a uniform slurry. Disk-shaped electrodes, approximately 12 mm in diameter, were produced using a stainless-steel mesh current collector. The electrodes had a thickness of 145 ± 10 μm. Each electrode contained an active material loading of 10 ± 1 mg cm^2^.^36,37^ The studies were performed in 6 M KOH and 0.5 M Na_2_SO_4_.

Cyclic voltammograms (CVs) and galvanostatic charge–discharge (GCD) plots were recorded across the potential range of −1 to 0.2 V vs Ag/AgCl. The scan rates and current density (*j*) ranged from 100 to 1 mV s^–1^ and 0.1 to 10 A g^–1^, respectively. *C*_sp_ values were determined using eq 1:^38^

1 $$C_{sp}=\frac{A}{2m\nu \times \Delta V}$$

where *A* represents the area enclosed by the CV, *m* refers to the weight of the active material, ν signifies the scan rate, and Δ*V* indicates the potential difference.^36^

The home-built symmetrical supercapacitor was composed of a two-electrode-cell configuration. A porous membrane made of a paper filter acted as the separator, while stainless-steel bars functioned as current collectors. The supercapacitor underwent testing with both aqueous and organic electrolytes, specifically 6 M KOH, 0.5 M Na_2_SO_4_, and 1 M NaClO_4_–(EC|PC). The organic electrolyte of 1 M NaClO_4_ was created with a weight ratio of 1:1 using a mixture of ethylene carbonate and propylene carbonate (EC|PC).

CVs were recorded under scan rates ranging between 100 and 1 mV s^–1^ across various potentials, while GCD tests were conducted at various potential windows with *j* values within the 0.1 to 10 A g^–1^ interval. Moreover, CVs at a scan rate of 20 mV s^–1^ assessing different maximum potential limits were acquired.

The *C*_sp_ values of the symmetric supercapacitor were evaluated using eq 2:^38^

2 $$C_{sp}=\frac{2A}{m\nu \Delta V}$$

where 2 indicates the configuration of two electrodes, while the other terms maintain the same definitions as those in eq 1.

Percentage of capacitance retention (% C_R_), energy density (*E*_D_), and power density (*P*_D_) were calculated using equations^34^

3 $$\%\;C_{R}=\frac{C_{F}}{C_{I}}\times 100$$

4 $$E_{D}=\frac{1}{8}C_{sp}V^{2}$$

5 $$P_{D}=\frac{E_{D}}{\Delta t}$$

where *C*_F_ and *C*_I_ are the final and initial capacitance, respectively, *V* ^2^ is the square of the operating voltage window, and Δ*t* is the discharge time.

Measurements using electrochemical impedance spectroscopy (EIS) were conducted in both three- and two-electrode configurations over a frequency range of 100 kHz to 10 mHz. The AC voltage applied was 5 mV. The effective capacitance (*C*_eff_) of a constant phase element (CPE) within a parallel R-CPE configuration, the transformation of CPE into a capacitance (*C*), as well as Warburg element impedance were determined using the procedure detailed in ref 34.

## 3 Results and Discussion

The chemical composition of GP, GPH, and GPB using EDS is detailed in Table 1. GP is composed of 31.14% C and 25.54% O (wt %), in addition to elements such as S, Ca, Si, and Fe, alongside portions of Mg and K. GPH exhibits similarities compared to GP, with variations in the concentration of the elements as a result of the hydrothermal treatment.

**Table 1:** Elemental Analysis of GP, GPH, and GPB^a^

material	C (wt %)	O (wt %)	K (wt %)	S (wt %)	Ca (wt %)	Mg (wt %)	Si (wt %)	Fe (wt %)	Zn (wt %)	Cl (wt %)
GP	31.14	25.54	0.77	3.56	19.44	0.47	8.22	4.74	nd	nd
GPH	31.70	20.91	1.22	3.30	7.93	0.30	15.89	8.53	nd	nd
GPB	41.90	21.83	0.17	1.98	1.34	0.39	9.60	2.94	13.06	0.18

a nd: not detected.

Meanwhile, GPB has the same elements as GPH but with notable changes in concentration. For example, the C and O contents are higher, whereas those of K, S, Si, and Fe are lower at the former. The Fe, K, and S elements can contribute to enhancing the electrochemical activity of GPB and its performance for supercapacitor applications. In addition, Si (or SiO_2_) can improve its electrochemical stability.^39^ The presence of Zn and Cl is attributed to the use of ZnCl_2_ as an activator.

Figure 1 illustrates the XRD patterns of GP, GPH, and GPB. GP shows (001), (002), and (200) reflections corresponding to planes at 2θ = 14.43, 30.47, and 37.57° , respectively, ascribed to the Ca–Al–Si compounds along with the peaks at 2θ = 21.68, 24.2, and 42.86° attributed to the (002), (110), and (004) planes of CaCO_3_ (JCPDS 00-013-0192), respectively.^40–42^ Furthermore, peaks at 29.07, 32.4, 40.36, and 49.84° are attributed to the (111), (200), (211), and (221) planes of FeS_2_ (JCPDS 00-002-1366), respectively. Moreover, peaks at 20.16, 22.93, 46.36, and 48.15° are attributed to the (220), (004), (218), and (185) planes of SiO_2_ (JCPDS 00-003-0218), respectively.^43^

**Figure 1:**
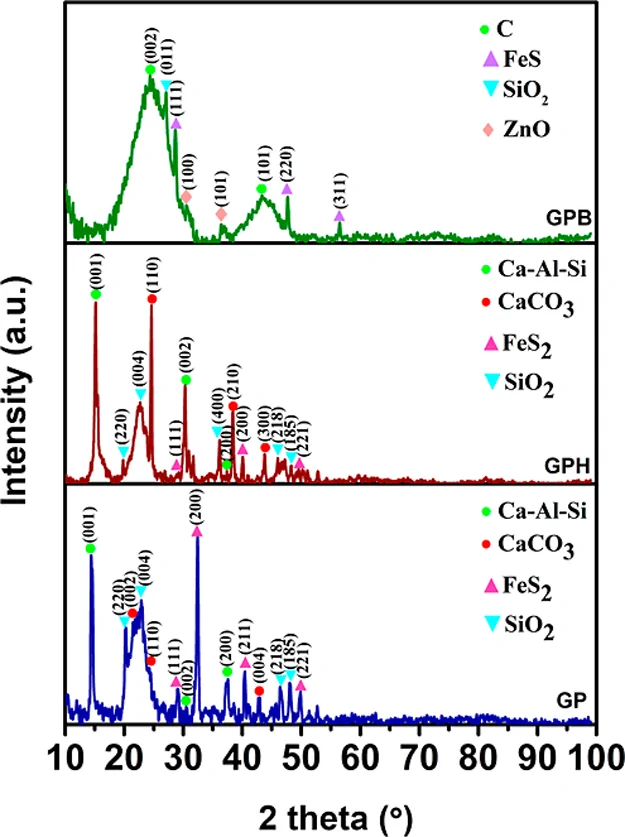
XRD patterns of GP, GPH, and GPB.

GPH shows similar features, with the (001), (002), and (200) reflections ascribed to Ca–Al–Si planes at 2θ = 15.14, 30.32, and 37.42°, respectively. It also has peaks at 2θ = 24.62, 38.40, and 43.84° ascribed to the (110), (210), and (300) planes of CaCO_3_, respectively.^44^ The reflections at 2θ = 28.93, 40.07, and 49.55° are ascribed to the FeS_2_(111), (200), and (221) planes, respectively. Even more, the peaks at 19.74, 22.66, 36.03, 46.07, and 48.30° are attributed to the (220), (004), (400), (218), and (185) planes of SiO_2_, respectively.

Meanwhile, GPB shows peaks at 24.32 and 43.42° assigned to the (002) and (101) planes of C (JCPDS 00-003-0401), respectively, suggesting that it has an amorphous nature.^43,45^ The spatial Ca–O correlation (EDS composition with low Ca concentration) and the absence of Ca peaks in the GPB pattern suggest that these phases are thermolabile and are eliminated during pyrolysis/activation with ZnCl_2_.^42,46^ Furthermore, peaks at 28.66, 47.61, and 56.53° are attributed to the (111), (220), and (311) planes of FeS (JCPDS 00-023-1123), respectively.^47^ The peak at 27.11° has been assigned to the (011) plane of SiO_2_ (JCPDS 00-086-1561). Additionally, peaks at 30.47 and 36.44° are ascribed to the planes (100) and (101) of ZnO (JCPDS 00-005-0664), respectively, which arise due to the chemical activation.^48^

Figure S1 (Supporting Information, SI) shows the deconvoluted Raman spectrum of GPB.^34^ Two peaks emerge at 1326 and 1598 cm^–1^, attributed to D and G bands, respectively. The D band is associated with the disruption in carbon atoms, resulting in structural disorder and defects, whereas the G band is attributed to the stretching vibrations of unsaturated carbon.^49^ The *I*_D_/*I*_G_ ratio of GPB of 1.04 indicates that it has a low degree of imperfections. Two additional peaks attributed to the D* and D′ bands are observed at 1192 and 1492 cm^–1^, respectively. The D* band arises from the breathing modes of *k*-point phonons in sp^2^-hybridized carbon domains, indicative of lattice defects such as vacancies, edge dislocations, and structural discontinuities. The D′ band reflects disordered graphitic regions with distorted symmetry, likely originating from residual heteroatoms or incomplete carbonization.^50^

Figure S2a exhibits an FESEM micrograph of GP with a morphology of large, nonhomogeneous, and uneven particles.^51^ Figure S2b shows a micrograph of GPH having clusters of relatively smaller porous particles when compared to GP. Figure S2c,d shows images of GPB at two magnifications. Its morphology is that of large smooth particles alongside small porous clusters similar to those at the GPH. Hydrothermal treatment and pyrolysis with ZnCl_2_ as an activator may have the effect of modifying the porosity of GP, leading toward the progress of micromesopores.^52^ A porous structure increases the surface area of carbon materials, hence improving their ability as an electrode in supercapacitors.^53^

Figure 2 displays the SEM chemical mapping of the GPB. Several of the elements listed in Table 1 exhibit homogeneous dispersion. The N heteroatom, not detected in the EDS analysis, as well as Si and S, shows a rather heterogeneous dispersion. It is worth noting that such heteroatoms have been shown to enhance biocarbon electrochemical performance in supercapacitor applications.^27,43^

**Figure 2:**
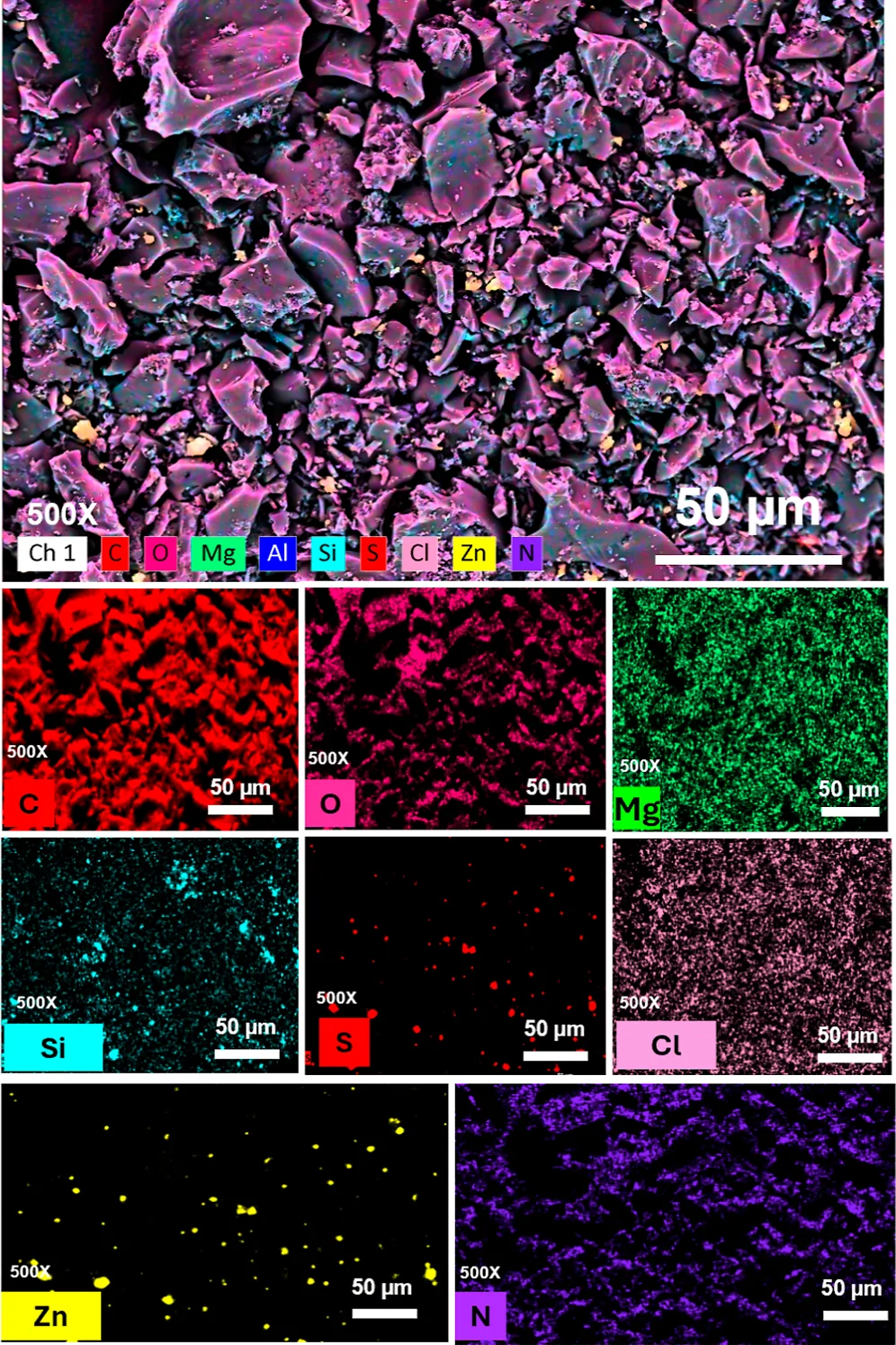
SEM images and elemental mapping of GPB.

The FTIR spectra of GP, GPH, and GPB (Figure 3) reveal significant shifts in the functional groups during each stage of the synthesis process. GP exhibits characteristic bands of polysaccharides, lignin, and other lignocellulosic biomass compounds.^54,55^ FTIR spectrum shows signals at 3336, 2918, 2845, 2161, 1618, 1418, 1325, 1150, 1100, 1015, 763, and 599 cm^–1^, ascribed to stretching of O–H bonds, symmetric and asymmetric C–H bonds, nitrile groups (C≡N), aromatic C=C bonds, C–H deformations, C–N bonds, C–O–C glycosidic bonds in cellulose/hemicellulose, C–O stretching in secondary alcohols or esters, asymmetric C–O bonds, and deformations of C–H in monosubstituted and ortho-disubstituted aromatic rings, respectively.^56,57^

**Figure 3:**
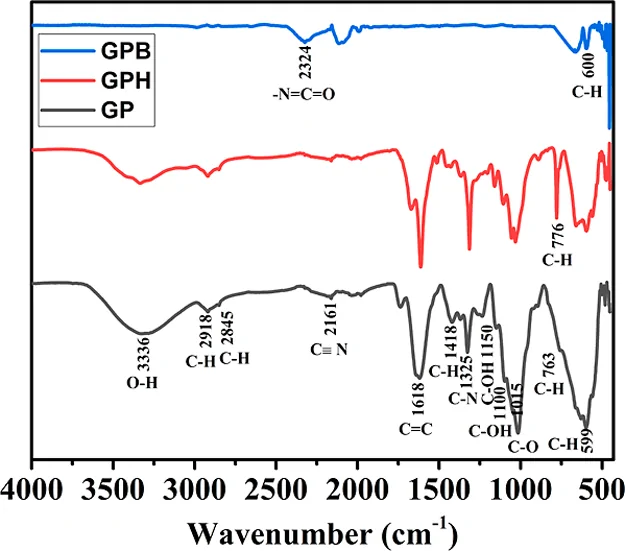
FTIR spectra of GP, GPH, and GPB.

GPH shows significant intensity changes in some bands compared to GP, suggesting a reorganization and concentration of certain structures during the hydrothermal treatment. Additionally, the out-of-plane deformation band of C–H bonds in monosubstituted aromatic rings undergoes a shift from 763 cm^–1^ (GP) to 776 cm^–1^ (GPH). This displacement reflects modifications in the electronic environment of aromatic systems, likely due to enhanced conjugation, steric effects, or the introduction of electron-withdrawing substituents during condensation and repolymerization reactions.^58–60^ In contrast, the band at 599 cm^–1^, related to out-of-plane C–H deformations in ortho-disubstituted aromatic rings, decreases in intensity compared to GP, which may be attributed to the partial degradation of such structures during the hydrothermal process.

In GPB, most bands disappear, indicating the transformation and condensation of complex chemical structures during pyrolysis. Dehydration, dehydrogenation, and condensation reactions lead to the formation of more ordered carbon structures, such as graphite-like domains.^61,62^

The emergence of a band at 2324 cm^–1^, absent in GP and GPH, suggests the formation of isocyanate (–N=C=O) groups, likely originating from nitrogen recombination during thermal degradation. The absence of the band at 763–776 cm^–1^ indicates that graphite structures restrict such vibrations, suggesting increased carbon order and condensation.^63^ The decline in the intensity of C–H bonds at 600 cm^–1^ indicates the degradation of ortho-disubstituted aromatic structures, contributing to a more homogeneous and ordered material. Overall, these changes reflect the conversion of complex structures into a more ordered and graphitic carbon material because of pyrolysis.

Figure S3a shows the N_2_ adsorption–desorption isotherm of GPB, having a IV (a) type behavior.^64,65^ Moreover, it shows characteristics of micromesoporous GPBs with an H4 hysteresis loop.^66,67^ Meanwhile, Figure S3b illustrates the pore distribution and pore volume curves of GPB, exhibiting micro- and mesopores varying from 3 to 6 nm, showing the hierarchical pore structure of GPB. The pore volume and SSA of GPB have been obtained using the BET method as 0.32 cm^3^/g and 1240.7 m^2^ g^–1^, respectively, with a mean pore size of 3.9 nm. Thus, the pyrolysis treatment produces a GPB with a high SSA and a mesoporous structure. Such features enhance the ion transfer at the electrolyte/electrode interface, reduce transport pathways, and typically enhance electrochemical performance.^68,69^

Figure 4 shows the XPS high-resolution spectra of GPB in the (a) C 1s, (b) O 1s, and (c) N 1s regions. Table 2 provides a list of parameters and data derived from their deconvolution. GPB exhibits multiple peaks at different binding energies (BE) in the C 1s region, attributed to different carbon chemical species: C=C (sp^2^), C–C (sp^3^), C–O/C–N=O, C=O/O=C–N, O–C=O, and a satellite signal associated with π–π* transitions in aromatic rings (BE = 284.8, 285.64, 286.56, 287.55, 289.25, and 291.07 eV, respectively).^70,71^

**Figure 4:**
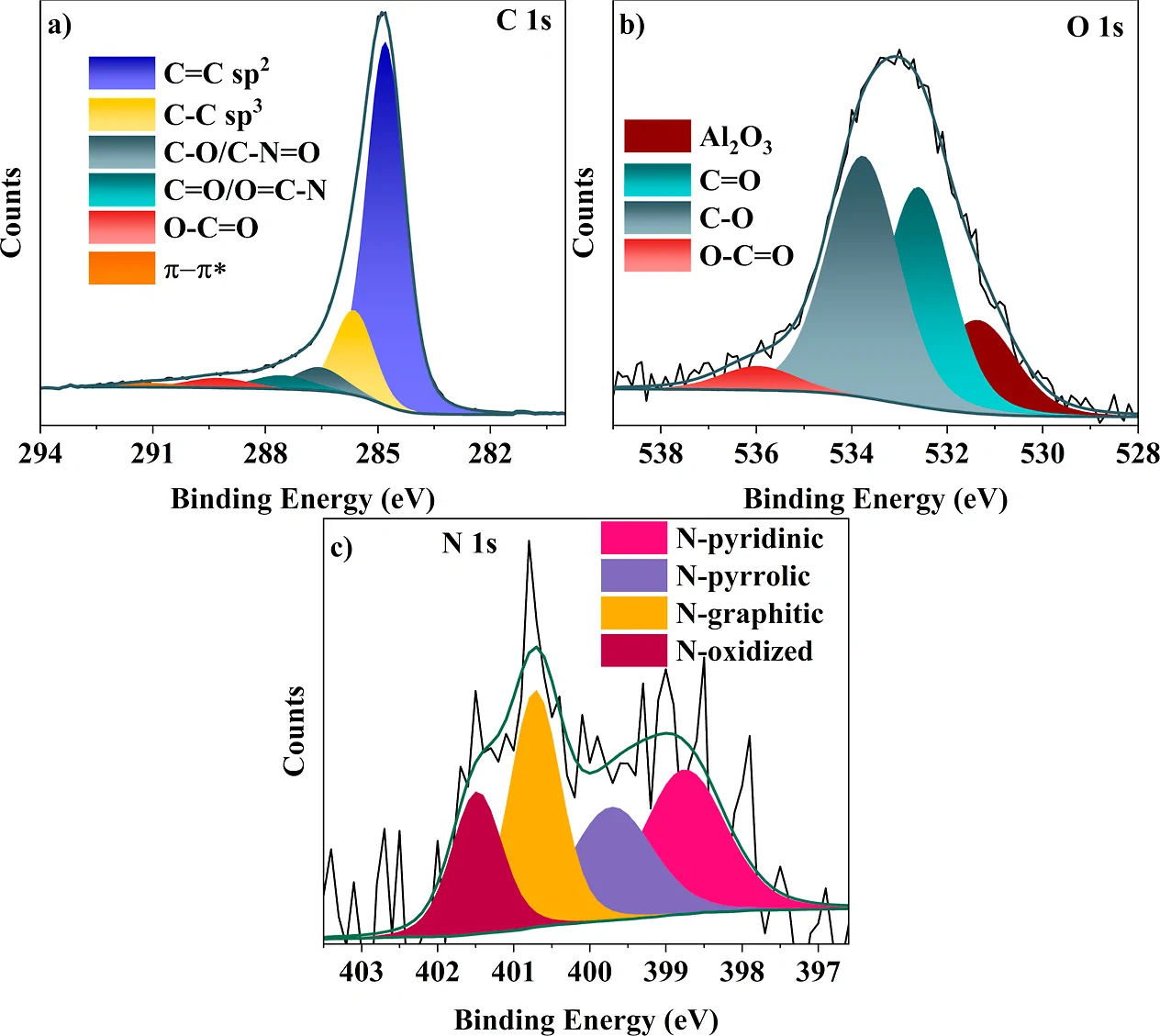
High-resolution XPS spectra of GPB in (a) C 1s, (b) O 1s, and (c) N 1s regions.

**Table 2:** Deconvoluted XPS Parameters of GPB

elements	state	species	BE (eV)	composition (%)
C	C 1s	C=C sp^2^	284.80	69.46
	C 1s	C–C sp^3^	285.64	15.93
	C 1s	C–O	286.56	5.88
	C 1s	C=O	287.55	4.15
	C 1s	O–C=O	289.26	3.18
	C 1s	π–π*	291.07	1.41
O	O 1s	Al_2_O_3_	531.35	21.38
	O 1s	C=O	532.59	21.81
	O 1s	C–O	533.76	14.66
	O 1s	O–C=O	535.96	2.99
N	N 1s	N-pyridinic	398.76	23.37
	N 1s	N-pyrrolic	399.71	17.47
	N 1s	N-graphitic	400.72	36.41
	N 1s	N-oxidized	401.48	22.75

The O 1s region of GPB shows peaks attributed to the presence of Al_2_O_3_, C=O, C–O, and O–C=O (BE = 531.35, 532.96, 533.76, and 535.96 eV, respectively).^44^

In the N 1s region, contributions from nitrogen in different configurations are identified: pyridinic, pyrrolic, N-graphitic, and N-oxidized (BE = 398.76, 399.71, 400.72, and 401.48 eV, respectively).^72^ The inherent N-species (pyridinic and pyrrolic) in GPB, derived from the natural N content of garlic peel,^73^ are critical for supercapacitor applications. The dominance of pyridinic-N, known for its electron-donating properties, suggests favorable pseudocapacitive behavior.^74,75^ This natural N incorporation circumvents the need for post-treatment doping, simplifying synthesis while maintaining performance.

Figure 5a shows the three electrode CVs of GPB in 6 M KOH, with scan rates varying from 100 to 1 mV s^–1^. The CVs show a quasi-rectangular shape, often seen in electric double-layer capacitors (EDLC), indicating a high-rate performance. These CVs showcase capacitive behavior, with high specific capacitance, which allows for efficient energy storage via fast charging and discharging.^76^ In order to determine EDLC and pseudocapacitive contributions to the electrochemical behavior of GPB, considering the presence of N- and O-species, analysis by the Dunn’s method has been performed with data from Figure 5a. The results are shown in the Supporting Information file and summarized in Table S1. As can be seen, between 70 and 90% of the carbon active sites contributed to EDLC behavior. Thus, even though not observed in the CVs of Figure 5 by means of redox pairs, it may be likely that, to some extent, there could be pseudocapacitance contributions by those species in GPB, of which the N heteroatom total content accounts for ca. 0.5 atom % in the biocarbon.

**Figure 5:**
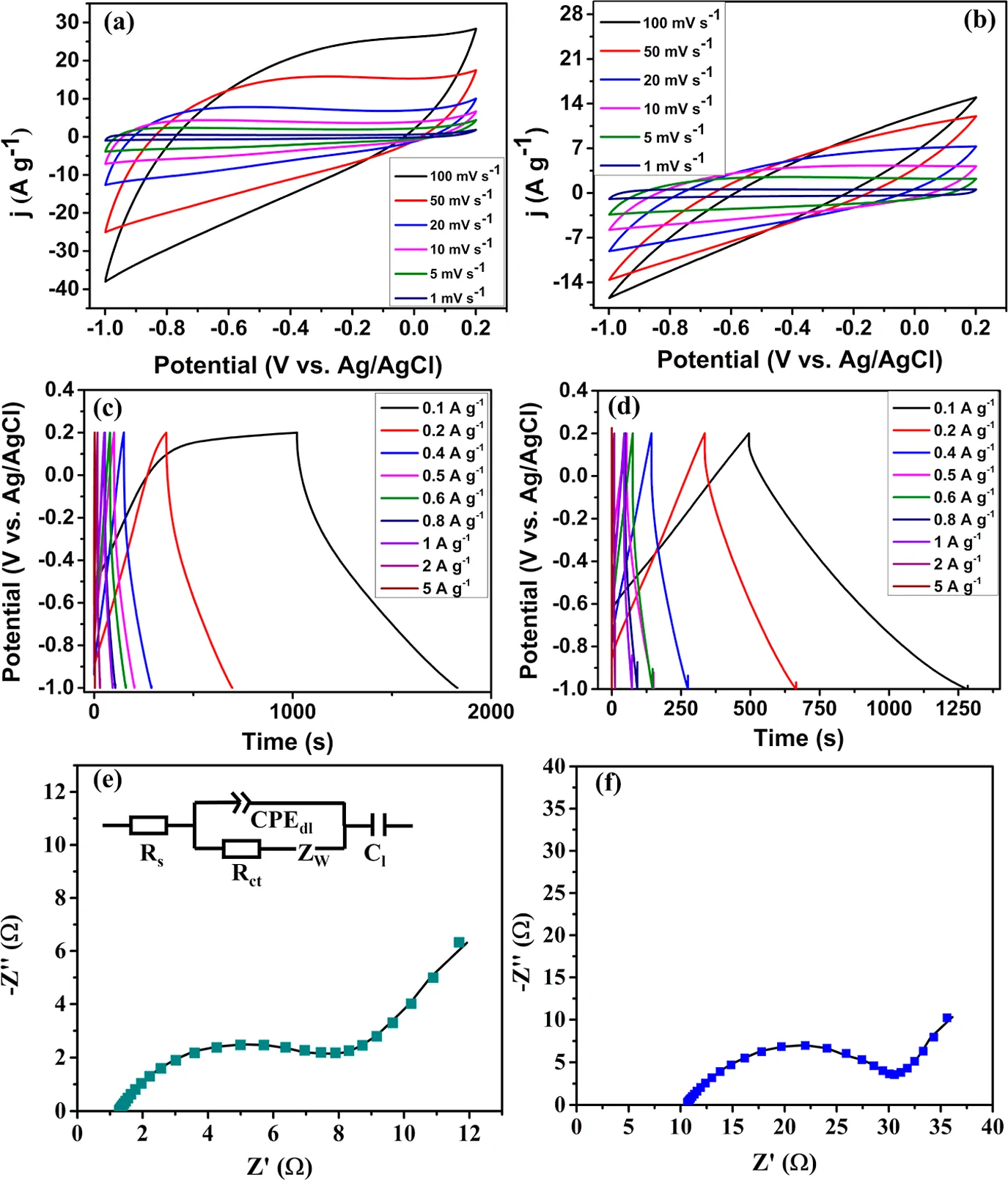
Electrochemical testing of GPB in a three-electrodes setup. (a, b) CVs at a scan rate ranging from 100 to 1 mV s^–1^ within the −1.0–0.2 V vs Ag/AgCl potential range. (c, d) GCD curves at *j* values from 0.1 to 5 A g^–1^. (e, f) Nyquist plots showing the equivalent circuit as the inset in (e). Electrolytes: (a, c, e) 6 M KOH; (b, d, f) 0.5 M Na_2_SO_4_.

Figure 5b CVs at the same scan rates for 0.5 M Na_2_SO_4_, having a distorted asymmetric rectangular shape. However, it is observed that the slope of the CV increases as the scan rate becomes higher, related to a fast decline in capacitive behavior due to incomplete formation of the EDL.^77^ This performance can result in nonideal capacitance behavior, which affects the specific capacitance and overall performance of the supercapacitor.^78^

Figure 5c,d GCD curves of GPB at different current densities ranging from 0.1 to 5 A g^–1^ in KOH and Na_2_SO_4_, respectively. In the former, GPB shows a symmetrical triangular curve at *j* values from 0.2 to 5 A g^–1^ without any potential drop, demonstrating a nonfaradaic reaction with low internal resistance. The output is an ideal capacitive behavior with high electrochemical reversibility. Meanwhile, at 0.1 A g^–1^, it shows an asymmetrical curve attributed to irreversibilities due to slow transfer at the electrode–electrolyte interface.^79,80^

In the latter electrolyte, the GCD curves at all *j* values have a nearly triangular shape. This shows ideal capacitive behavior, which suggests good reversibility, which is determined by the kinetics of ion transport and storage processes occurring at the electrode/electrolyte interface.^31^

Figure 5e,f Nyquist plots of GPB, KOH, and Na_2_SO_4_, respectively. Inset in (e) shows the fitting equivalent circuit: *R*_s_, solution (or impregnated separator) resistance; *R*_ct_, charge-transfer resistance at the electrode/electrolyte interface; CPE_dl_, nonideal capacitive behavior due to surface heterogeneity and pore-size distribution; *Z*_w_, Warburg impedance element; and *C*_1_, limit capacitance associated with the ion diffusion in the porous electrode structure.^81^ The fitting values are depicted in Table S2.

In 6 M KOH, the low *R*_s_ (1.27 Ω) confirms the high ionic conductivity of this concentrated alkaline medium, while the moderate *R*_ct_ (6.19 Ω) indicates relatively facile ion exchange at the electrode–electrolyte interface,^25,82^ contributing to the reversible behavior observed in Figure 5c. The CPE_dl_ value highlights the presence of a large electrochemically active surface area, and the *C*_1_ interfacial contact capacitance further reflects the highly porous structure of GPB.^25^ The Warburg impedance remains moderate, suggesting effective ion diffusion at low frequencies.

In contrast, *R*_s_ in 0.5 M Na_2_SO_4_ (10.57 Ω) is significantly higher than in KOH, underscoring the lower ionic conductivity of the sulfate-based electrolyte.^83,84^ Moreover, the larger *R*_ct_ (21.36 Ω) also corroborates a slower ion exchange process, as evidenced by the larger semiarc noted in the Nyquist plot. Despite the smaller CPE_dl_ value, the *C*_1_ interfacial capacitance is notably higher, possibly indicating additional adsorption processes on the electrode surface.^85^ Meanwhile, the Warburg element points to more restricted ion diffusion within the porous network under these sulfate conditions compared to KOH.

Figure 6a shows the *C*_sp_ values of GPB with various scan rates, determined using eq 1, in the two electrolytes using the three-electrode configuration. The higher *C*_sp_ obtained is 183.5 F g^–1^ in 6 M KOH at 1 mV s^–1^, compared to 168.7 F g^–1^ in 0.5 M Na_2_SO_4_. As the scan rate rises, the *C*_sp_ decreases, a phenomenon occurring because the active surface area is not fully used, resulting in less time for ions to diffuse into the porous structure.^86^ For instance, the *C*_sp_ value at 100 mV s^–1^ in KOH falls to 95.99 F g^–1^. The biocarbon exhibits a similar tendency of *C*_sp_ at various scan rates in 0.5 M Na_2_SO_4_, nevertheless with smaller values because of the limited ion diffusion and absorption of this electrolyte into the porous structure.^87^

**Figure 6:**
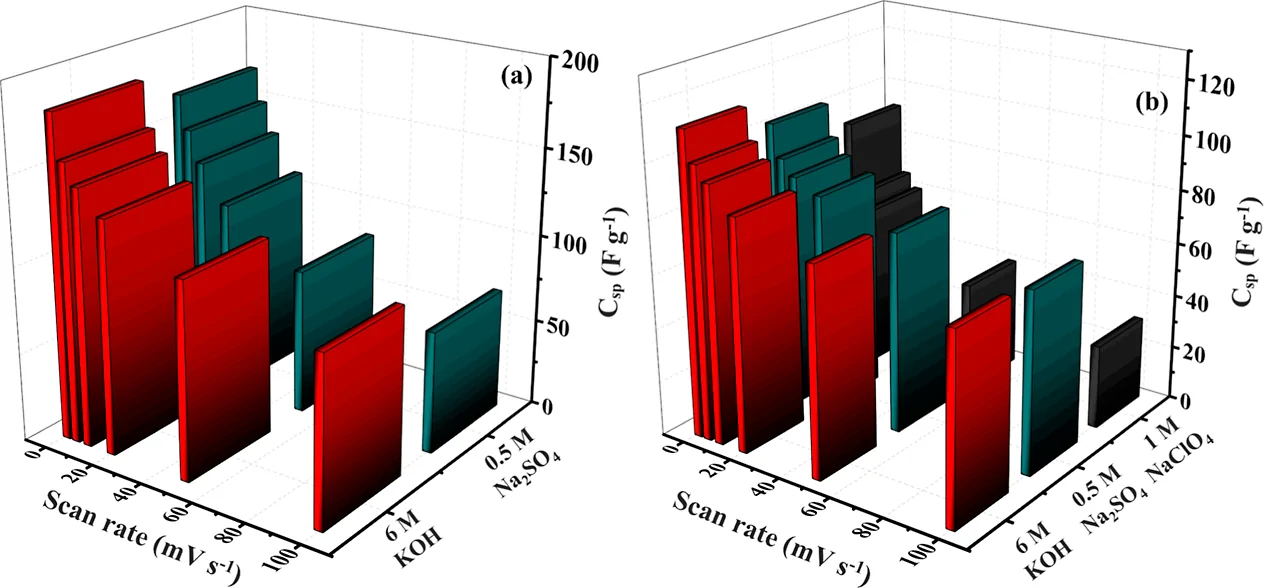
GPB *C*_sp_ values vs scan rates varying from 100 to 1 mV s^–1^ in (a) the three-electrode cell with 6 M KOH and 0.5 M Na_2_SO_4_ and (b) the symmetric supercapacitor with 6 M KOH, 0.5 M Na_2_SO_4_, and 1 M NaClO_4_–(EC|PC).

Figure 7a depicts the symmetrical supercapacitor CVs in 6 M KOH with scan rates varying from 100 to 1 mV s^–1^. The plots exhibit a notable rectangular shape without significant distortion despite the high scan rates, indicating a capacitive response resulting in the development of an EDLC. The potential window of 0–1 V in this electrolyte is restricted mainly by the thermal stability limit of water, which is 1.23 V, limiting irreversible reactions.^88^ Figure 7b depicts the CVs at various potential limits at 20 mV s^–1^ in the same electrolyte. The plots exhibit a nearly rectangular shape, with a slight *j* value increase, due to a good rate capability and ion transport at the electrode/electrolyte interface.^89,90^

**Figure 7:**
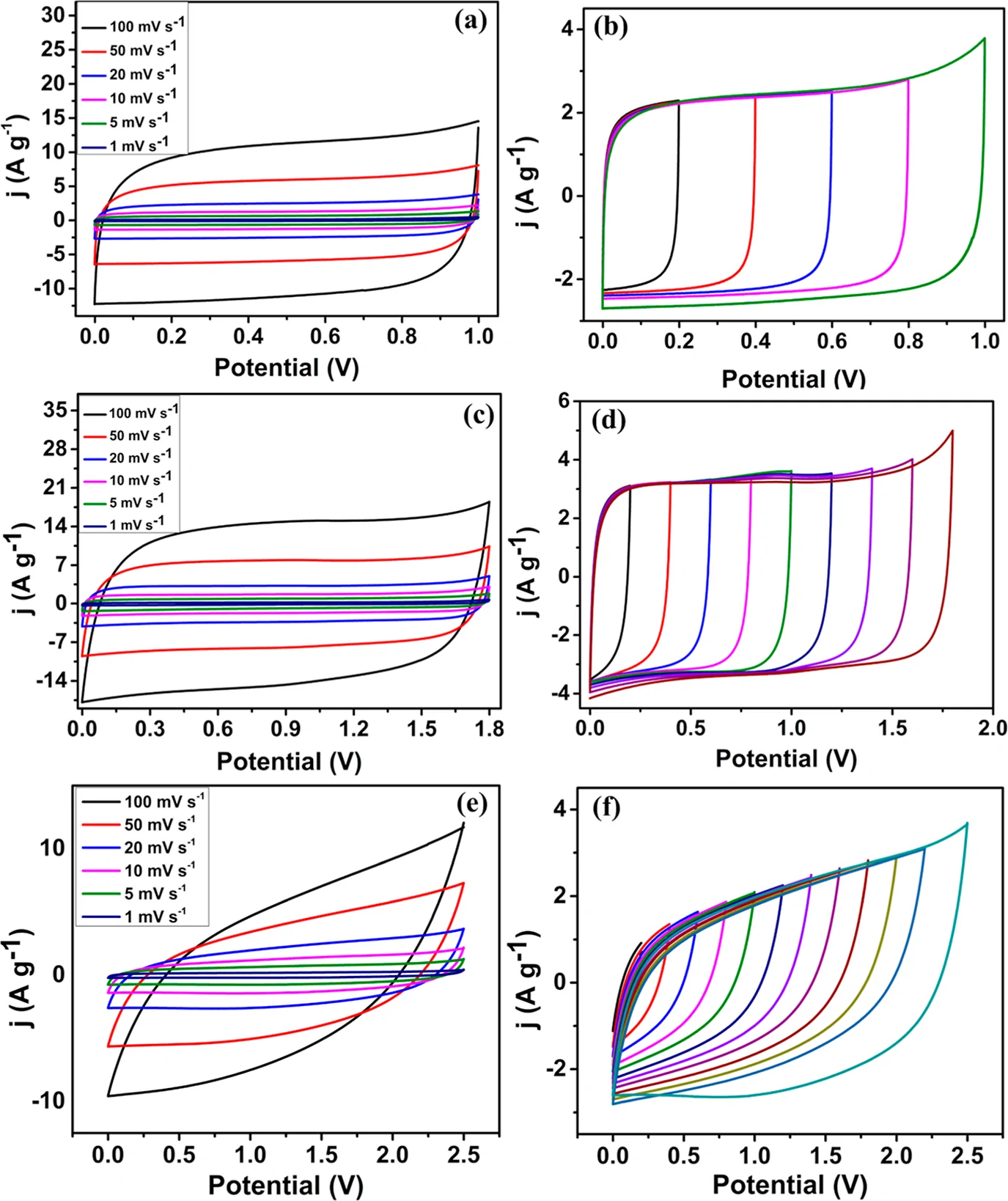
Symmetric supercapacitor testing of GPB. CVs at a scan rate ranging from 100 to 1 mV s^–1^ and at several potential limits at 20 mV s^–1^ in (a, b) 6 M KOH, (c, d) 0.5 M Na_2_SO_4_, and (e, f) 1 M NaClO_4_–(EC|PC).

Meanwhile, Figure 7c shows the CVs in 0.5 M Na_2_SO_4_ under the same condition as in (a). The CV curves show a quasi-rectangular shape throughout the 0–1.8 V potential window by fast ion diffusion and facile transport within the electrolyte/electrode, exhibiting capacitive behavior.^91^ In contrast to KOH, neutral Na_2_SO_4_ inhibits water decomposition, thereby enabling a broader potential range, nevertheless with apparently slower kinetics.^92^ The lack of significant pH fluctuations delays the onset of hydrogen evolution (HER) and oxygen evolution (OER) reactions, allowing for operation at relatively elevated voltage without increased degradation.^88^

Additionally, Figure 7d depicts the CVs at different potential limits at 20 mV s^–1^ in 0.5 M Na_2_SO_4_. The curves are quasi-rectangular, and when the potential exceeds 1.5 V, a little deviation in the CVs becomes visible. Also, there is an increase in *j* when the potential rises to 1.8 V, a peak that occurs because of oxygen evolution with the degradation of the electrolyte.^91,93^

In the meantime, Figure 7e depicts the CVs under the same conditions as in (a) in 1 M NaClO_4_–(EC|PC). The CV curves show a quasi-rectangular behavior demonstrating the presence of a distinct EDLC contribution. NaClO_4_–(EC|PC) offers the widest potential window of 0–2.5 V in the figure. Unlike KOH, NaClO_4_–(EC|PC) lacks water, an advantage that avoids HER and OER limitations, while its elevated breakdown voltage (∼2.5–3 V) facilitates high-energy storage.^94,95^

Investigations show that when the scan rate rises, the *j* values in cathodic and anodic sweeps likewise increase, demonstrating a good reversibility of the active materials.^96^ However, there is a fast charge transfer at both potential ends and distortion in the shape, indicating a slight decrease in capacitance and insufficient formation of an EDLC.^97,98^

Figure 7f CVs at 20 mV s^–1^ in NaClO_4_ at various potential limits. The optimal one has been determined from 0 to 2.5 V, i.e., the widest among the three electrolytes evaluated in the symmetric supercapacitor in Figure 7. Exceeding these potential restrictions is challenging since it may cause the electrolyte to decompose.^99^

The *C*_sp_ calculated from eq 2, based on the CVs of the symmetric supercapacitor operating with 6 M KOH, 0.5 M Na_2_SO_4_, and 1 M NaClO_4_–(EC|PC) in Figure 7, are 112.5, 103.2, and 99.2 F g^–1^ at 1 mV s^–1^, respectively. Lower scan rates provide improved interaction between GPB and the electrolyte, resulting in an enhanced diffusion of the electrolyte into the porous network.^100^ The results show that the *C*_sp_ in NaClO_4_ is not significantly lower than those in KOH and Na_2_SO_4_, the former having the advantage of sustaining a wider potential window, as demonstrated in Figure 7.

Figure 6b *C*_sp_ vs scan rate plot of the symmetric supercapacitor. It is seen that there is a decrease in *C*_sp_ with KOH and Na_2_SO_4_ electrolytes, from the values in the three-electrode configuration to those in the symmetric two-electrode supercapacitor. A decrease in *C*_sp_ is expected when a two-electrode device is tested, since internal series resistance may increase due to the contributions of 2 active working electrodes, 1 porous separator impregnated with electrolyte, and 2 current collectors. Such elements may have caused the decrease in *C*_sp_ seen in Figure 6b. Since a mass balancing has been performed between the 2 electrodes, this factor is discarded as a contributor of the mentioned *C*_sp_ decrease.

The GCD graphs of the symmetric supercapacitor at different current densities ranging from 0.1 to 10 A g^–1^ are shown in Figure 8a–c for the three different electrolytes. In KOH and Na_2_SO_4_, the charge–discharge profiles exhibit a modest change with a slight potential drop, indicating high Coulombic efficiency. This confirms the favorable electrochemical reversibility of supercapacitors. On the other hand, the profiles in NaClO_4_ are rather asymmetrical at all *j* values. Therefore, the charging takes a longer time than the discharging, leading to a slightly lower Coulombic efficiency compared to the two other electrolytes. This might be attributed to solution/electrode resistances and limited usage of the active mass in the electrode.^91^

**Figure 8:**
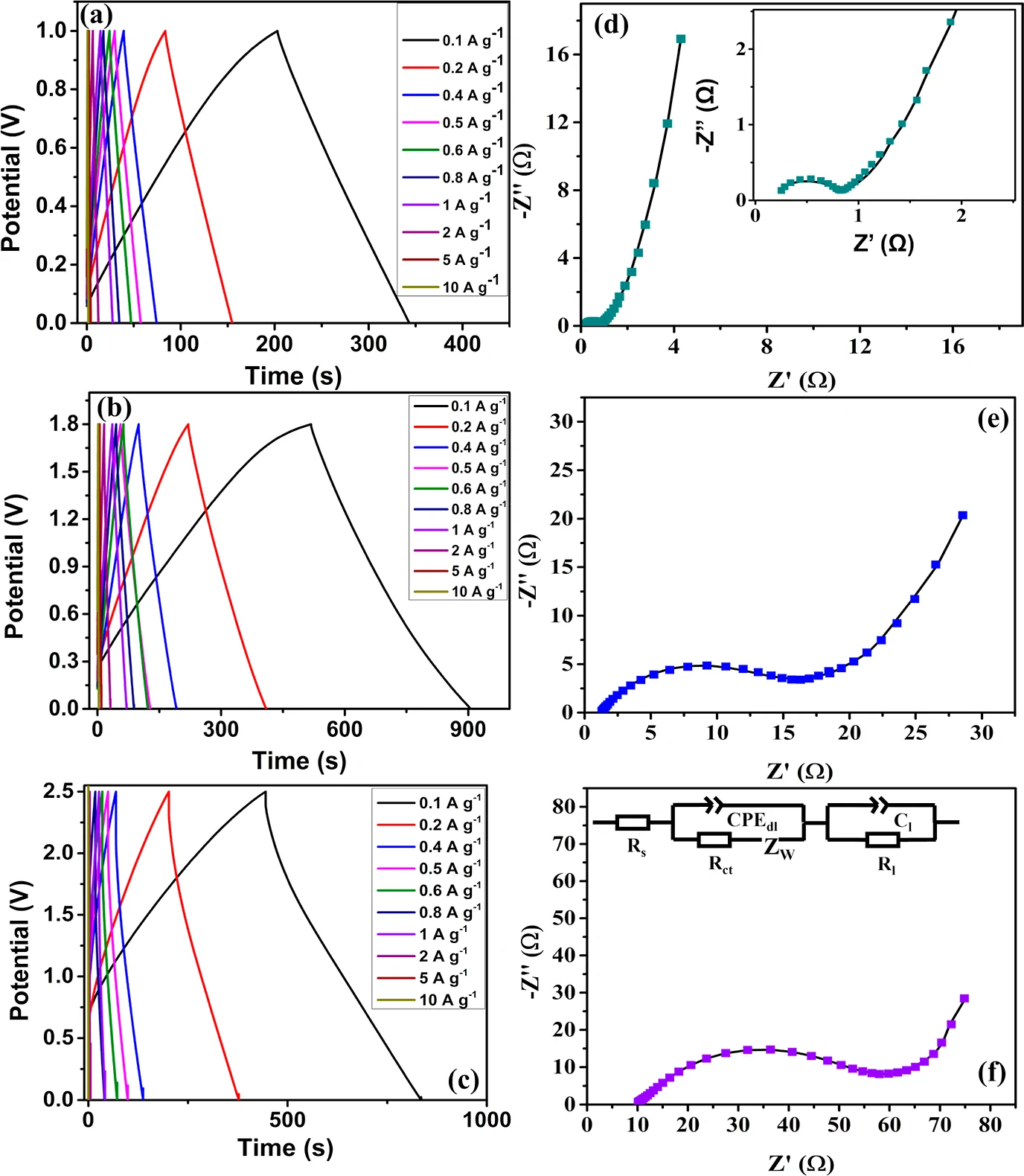
Symmetric supercapacitor testing of GPB. GCD plots recorded under current density values ranging from 0.1 to 10 A g^–1^ in (a) 6 M KOH, (b) 0.5 M Na_2_SO_4_, and (c) 1 M NaClO_4_–(EC|PC). (d–f) Corresponding Nyquist plots, with the equivalent circuit shown in (f).

Figure 8d–f EIS Nyquist plots of GPB in the symmetric supercapacitor with the different electrolytes. The equivalent circuit utilized for fitting is illustrated as an inset in Figure 8f, where *R*_l_ is the leakage or self-discharge resistance associated with the charge retention capacity of the electrode.^34^ Table S3 summarizes the fitted values of the elements.

In 6 M KOH, the very low *R*_s_ (0.185 Ω) confirms the excellent ionic conductivity of this strongly alkaline medium, while the *R*_ct_ (0.573 Ω) remains relatively small, facilitating rapid electrochemical reactions at the electrode interface.^101^ The low *C*_1_ (74.59 × 10^–6^ F) may be related to the large SSA of GPB, providing efficient ion access through its mesoporous network in KOH.^102^ Moreover, the *C*_1_ value indicates that the porous electrode structure contributes significantly to charge storage, while the moderate *Z*_w_ (12.31 Ω) reflects effective ion diffusion at low frequencies.^103^

In 0.5 M Na_2_SO_4_, *R*_s_ (1.23 Ω) is higher than in KOH, indicating reduced ionic conductivity due to its lower concentration and different ionic species. Despite the small *R*_ct_ (16.0 Ω) reported, the *Z*_w_ element (57.14 Ω) is substantially larger than in KOH, suggesting that distant ion diffusion becomes more restrictive in this electrolyte.^104^ The *C*_1_ (0.0662 × 10^–3^) is higher than in KOH, the ion sizes of Na^+^ and SO_4_^2–^ are larger, resulting in reduced double layer thickness, potentially indicating a less accessible surface for fast ion adsorption. Meanwhile, *C*_1_ is lower compared to KOH, in line with the diminished conductivity and less favorable charge accommodation compared to the alkaline medium.^105^

In 1 M NaClO_4_, *R*_s_ (10.1 Ω) and especially *R*_ct_ (50.23 Ω), are significantly higher than in both KOH and Na_2_SO_4_, confirming that the organic-based electrolyte has more challenging conditions for rapid charge transport.^106^ The low resistance in this system arises from related ion mobility, defects in the solid electrolyte interphase (SEI), and the presence of impurities. However, the *C*_1_ (3.788 × 10^–5^) is small, suggesting more limited ion accessibility at the electrode surface, even though *C*_1_ is of the same order as in KOH. The *Z*_w_ element (52.35 Ω) underscores further mass transport limitations, which, together with the high *R*_ct_, explain the lower *C*_sp_ value in the organic electrolyte compared to the aqueous electrolytes.^107^ Overall, the *R*_l_ values are low, indicating reduced charge retention or enhanced parasitic Faradaic processes in the three electrolytes, the latter most likely originating from a pseudocapacitance contribution of the N-species, as discussed above.^34,108^

The % *C*_R_ plots of the symmetric supercapacitor after undergoing 10,000 cycles at 100 mV s^–1^ are shown in Figure 9a–c. The insets provide additional information by illustrating the CVs during the 1st and 10,000th cycles. The % *C*_R_ in KOH is around 91.91%, ascribed to fast ion transport between the electrodes. This value surpasses that of Na_2_SO_4_, at which its neutral pH and lower gas evolution reduce corrosive side reactions compared to KOH; however, gradual oxidation at 1.8 V results in a lower capacitance.^109^ Meanwhile, the % *C*_R_ value of NaClO_4_ is the lowest. Organic electrolytes exhibit degradation over multiple cycles, resulting in the formation of an SEI that elevates internal resistances without causing noticeable differences in the CV profile.^94,110^ Thus, the % *C*_R_ of the supercapacitor with NaClO_4_ drops to 78.38%.

**Figure 9:**
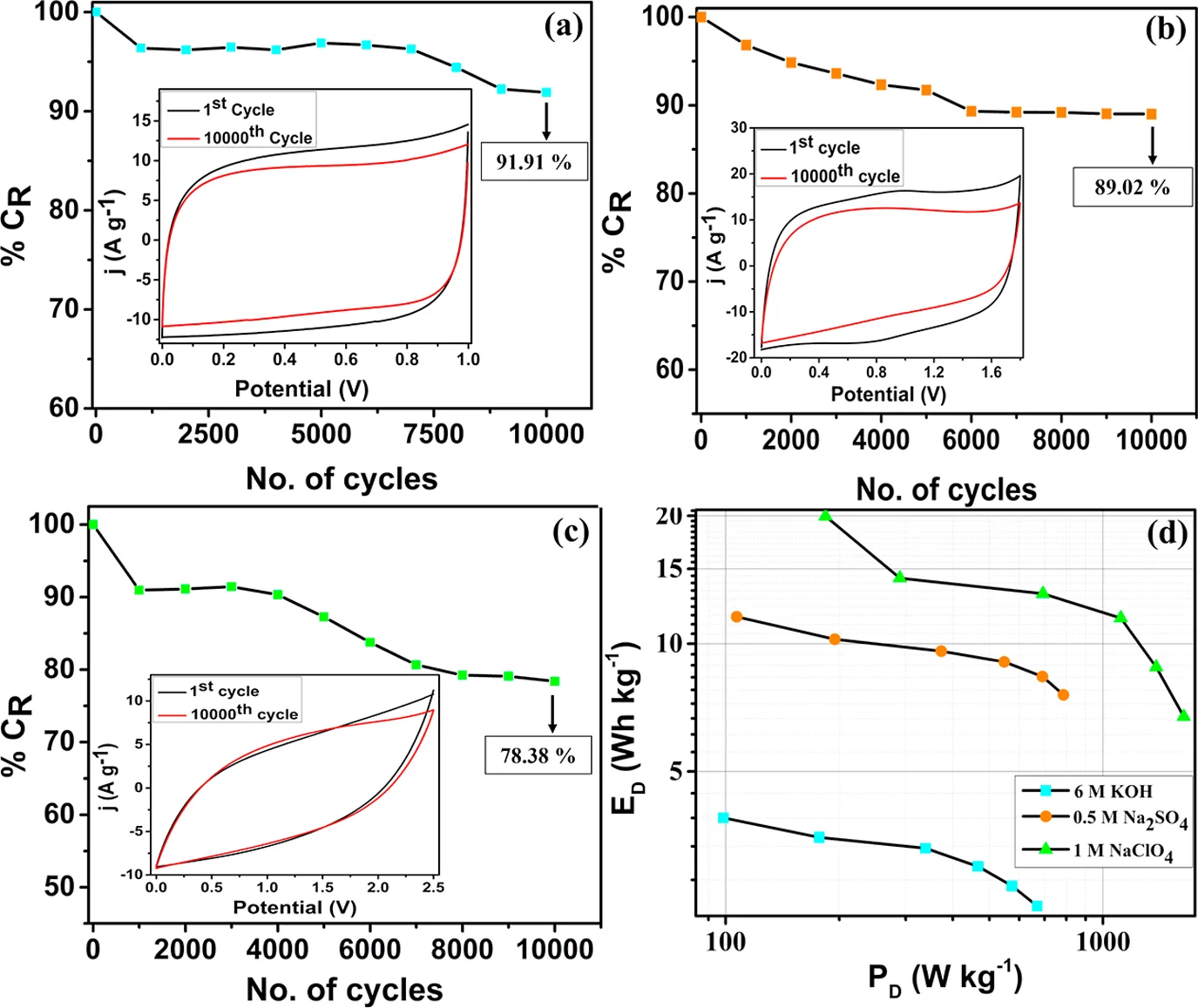
(a–c) % *C*_R_ plots at the scan rate of 100 mV s^–1^. Insets: CVs in the 1st and 10,000th cycles. Electrolytes: 6 M KOH, 0.5 M Na_2_SO_4_, and 1 M NaClO_4_–(EC|PC), respectively. (d) Ragone plot of the symmetric supercapacitor with the three electrolytes.

Figure 9d Ragone plots of the supercapacitor using the three electrolytes. The maximum *E*_D_ falls in the following order: 19.9 > 11.57 > 3.89 Wh kg^–1^. These values correspond to *P*_D_ values of 183.9, 107.0, and 98.75 W kg^–1^ in NaClO_4_, Na_2_SO_4_, and KOH, respectively. The organic NaClO_4_–(EC|PC) electrolyte shows a wider potential window in Figure 7 (2.5 V) than Na_2_SO_4_ (1.8 V) and KOH (1.0 V), which allows it to achieve higher *E*_D_ and *P*_D_ values compared to the aqueous electrolytes, consistent with eq 4.

Hence, 1 M NaClO_4_ shows ∼5-fold and 2-fold increases in *E*_D_ and *P*_D_, respectively, compared to 6 M KOH. These features highlight the advantageous GPB/NaClO_4_ configuration that improves *E*_D_ and *P*_D_ outputs in a symmetric supercapacitor, which originate primarily from the wider voltage window allowed by the organic electrolyte. In contrast, the KOH electrolyte provides higher *C*_sp_, lower *R*_s_ and *R*_ct_, and better cycling stability. Therefore, the observed differences arise from the combined effects of both the electrode material and electrolyte properties.

Several aspects of the GPB performance with the three electrolytes, in 3- and 2-electrodes systems, should be discussed. The textural properties of GPB show that SSA = 1240.7 m^2^ g^–1^ and mean pore diameter = 3.96 nm, confirming a micro–mesoporous structure. Additionally, the normalized capacitance per unit surface area (*C*_A_) of GPB has been determined with eq S1 shown in the SI file. As can be seen, *C*_A_ = 14.8 μF cm^–2^ in KOH (data from Figure 5a). This value may be considered in the lower end of commonly reported values of about 15–18 μF cm^–2^ for aqueous electrolytes.^111,112^

Therefore, it is suggested that the electrochemical response of GPB is consistent with an important fraction of BET SSA being electrochemically available, while some other portions remain not easily accessible to the electrolyte. Even though the SSA is high, the *C*_sp_ achieved by GPB in the different electrolytes is relatively low. This behavior suggests that there may be failures within the interconnected micro- and mesoporous channels, which limit efficient electrolyte transport within some portions of the GPB network.

In a recent study, it has been reported that channel tortuosity is an important factor affecting negatively the performance of porous carbons in supercapacitors, and the limitations increase as the electrolyte cation becomes bigger.^113^ Even though more studies will be performed in future works on GP-derived biocarbons by our group, it is hypothesized that the relatively low *C*_sp_ values achieved in the electrolytes are due to a nonoptimized micromesoporous network. This is more clearly seen in the behavior of GPB in NaClO_4_ (the largest cation). Such textural issues may also be responsible for the low % *C*_R_ of the supercapacitor operating with such an organic electrolyte.^113^

An analysis has been conducted to underline relevant parameters of the GPB-based symmetric supercapacitor operating with the three electrolytes, resulting in the radar chart in Figure 10.^114^ Details of how the chart has been constructed are given in the SI. The radar chart exhibits the following relevant metrics: *P*_D_, *E*_D_, *C*_sp_, voltage window, *R*_ct_, % *C*_R_, and stability cycles. Highlights of electrolyte performance with GPB as active material depicted in Table S4.

**Figure 10:**
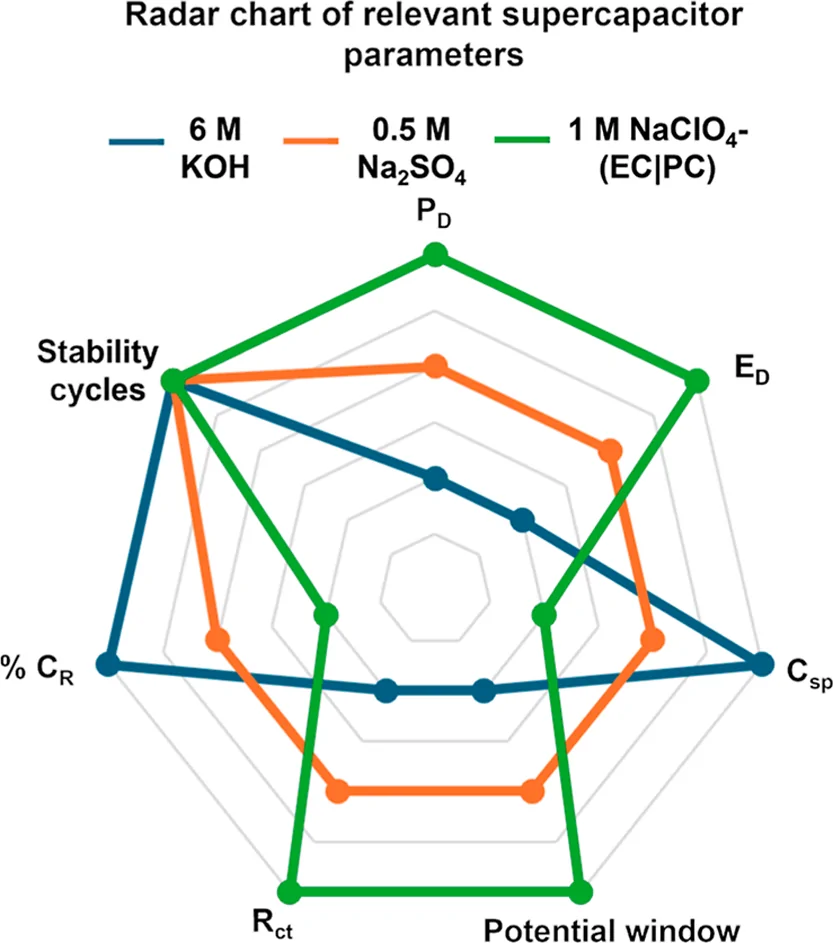
Radar chart of relevant metrics of the symmetric supercapacitor operating with the three electrolytes and GPB as the active material.

While the highly conductive 6 M KOH shows good storage kinetics, exhibiting the lowest *R*_ct_ and highest *C*_sp_, alongside important cyclic retention, it is fundamentally constrained by its narrow water stability limit.

Occupying a middle ground, neutral 0.5 M Na_2_SO_4_ provides a balanced compromise; its mitigated corrosivity and lack of significant pH fluctuations delay hydrogen and oxygen evolution, extending the voltage window to 1.8 V. Nevertheless, it is outperformed in every metric by one of the electrolytes.

Ultimately, the organic 1 M NaClO_4_–(EC|PC) footprint clearly expands the operating voltage window, reaching 2.5 V. Such voltage stability increases the *E*_D_ and *P*_D_ values achieved by the supercapacitor. Consequently, the organic system dictates the energy and power axes, delivering maximum values (19.9 Wh/kg and 183.9 W/kg, respectively). Because *E*_D_ scales quadratically according to eq 4, this thermodynamically advantageous expansion overcomes the electrolyte’s more restrictive ion diffusion, lower % *C*_R_, and higher *C*_sp_. Thus, the multifactorial chart visually rationalizes the selection of 1 M NaClO_4_–(EC|PC) as the optimal electrolyte for the home-fabricated GPB supercapacitor by mapping the critical trade-offs among thermodynamics, kinetics, and durability.

Therefore, in addition to the Ragone plot, the radar chart shows the superior performance of the GPB-based symmetric supercapacitor operating with NaClO_4_(EC|PC). However, it is acknowledged that several important parameters must be improved at the GPB/NaClO_4_ interface. Such further studies are beyond the scope of this work.

Additionally, Table S5 shows a comparison of the performance of GPB in the three electrolytes with biocarbons produced from garlic and other biomass resources. The GPB||GPB supercapacitor with the NaClO_4_–(EC|PC) compares adequately with systems like orange and cassava peel biocarbons in terms of *E*_D_ and, to some extent, cycling stability. Moreover, it is to be noted that the GPB||GPB supercapacitor with 0.5 M Na_2_SO_4_ achieves a higher *E*_D_ than orange peel with 0.5 M Na_2_SO_4_ (ref 10 in the SI file). However, GPB fails to be as good as other garlic-based biocarbons. The evaluation indicates that some parameters of the synthesis method could be modified, aiming to enhance the physicochemical features of GPB, nevertheless keeping the less aggressive conditions, for instance, acetic acid concentration during hydrothermal treatment and carbon-to-activator mass ratio during pyrolysis.

## 4 Conclusions

GPB with high SSA and a mesoporous structure was produced from the activation of GP biowaste. The thermal treatment produced a biocarbon with advantageous characteristics for supercapacitor applications, such as fast ion transfer at the electrolyte/electrode interface and short transport pathways.

Based on the evaluation with three electrolytes, KOH showed high ionic conductivity but the narrowest voltage window in the symmetric supercapacitor, leading to poor *E*_D_ and *P*_D_ values. Meanwhile, Na_2_SO_4_ and NaClO_4_ provided wider potential windows of 1.8 and 2.5 V, respectively, in the devices. Therefore, the *E*_D_ and *P*_D_ values with the organic electrolyte were the highest (19.9 Wh kg^–1^ and 183.9 W kg^–1^, respectively). Nevertheless, it was acknowledged that this environment resulted in lower *C*_sp_ and % *C*_R_ values, with higher *R*_ct_ values.

Even though the *E*_D_ achieved by GPB in this study may be lower than previous reports with analogous garlic-waste-based biocarbons, in future works, some synthesis parameters in this novel process may be adjusted (e.g., acetic acid concentration during hydrothermal treatment and carbon-to-activator mass ratio during pyrolysis) to improve its physicochemical properties and therefore its electrochemical behavior.

The modification of parameters was also projected, considering the low yield (14.55%) of GPB, and intended to improve this important metric. On this issue, different garlic species can be evaluated, aiming to propose a more suitable, cost-effective process for the application of this biomass in supercapacitor technologies.

Hence, this study stated a conversion of abundant GP biowaste to GPB by a cost-effective process. The electrochemical performance of GPB demonstrated its potential as an alternative active electrode material for supercapacitors. Moreover, the study of different environments with GPB may serve the purpose of helping in the selection of the adequate electrolyte for specific symmetric supercapacitor applications, that is, higher *E*_D_ and *P*_D_ versus higher *C*_sp_.

## Supplementary Material



## Data Availability

The data underlying this study are available in the published article and its SI.

## Notes

Raman spectrum, FESEM micrographs, adsorption isotherm and pore diameter and pore volume distribution curves of GPB; Dunn’s analysis, calculation of *C*_A_, and EIS parameters from 3-electrodes and symmetric supercapacitor characterization; procedure for data normalization of radar chart, with highlights of electrolyte performance; and comparison of GPB behavior with garlic-based and other biomass-derived porous biocarbons in supercapacitor applications (PDF)
