# Supplementary material for: Porous Biocarbon Derived from Garlic Peel Biowaste for Symmetric Supercapacitor Applications: Electrochemical Performance in Aqueous and Organic Electrolytes
Source: ACS Omega. 2026 Sep 21;11(39):58836–50. doi: 10.1021/acsomega.6c03559 (PMC13647417; doi:10.1021/acsomega.6c03559)
Supplement: Supplementary file 1 [file ao6c03559_si_001.pdf]

Porous biocarbon derived from garlic peel biowaste for symmetric supercapacitor applications: electrochemical performance in aqueous and organic electrolytes

Santhosh Ravichandran [a], Manuel Octavio Fuentez-Torres [a], Ivonne Liliana Alonso-Lemus [b,*], Beatriz Escobar-Morales [c], Francisco Javier Rodríguez-Varela [a,d,*]

[a] Nanociencias y Nanotecnología, Cinvestav Saltillo, Av. Industria Metalúrgica 1062, Ramos Arizpe, Coahuila, C.P. 25900, México

[b] Secihti-Cinvestav Saltillo. Sustentabilidad de los Recursos Naturales y Energía. Av. Industria Metalúrgica 1062, Parque Industrial Ramos Arizpe. Ramos Arizpe, Coahuila, C.P. 25900, México

[c] Secihti, Centro de Investigación Científica de Yucatán, Unidad de Energía Renovable, Calle 43, No. 130 Col. Chuburná de Hidalgo, Mérida, Yucatán, C.P. 97200, México

[d] Sustentabilidad de los Recursos Naturales y Energía, Cinvestav Saltillo, Av. Industria Metalúrgica 1062, Ramos Arizpe, Coahuila, C.P. 25900, México

*Corresponding authors

E-mail address: ivonne.alonso@cinvestav.edu.mx; javier.varela@cinvestav.edu.mx

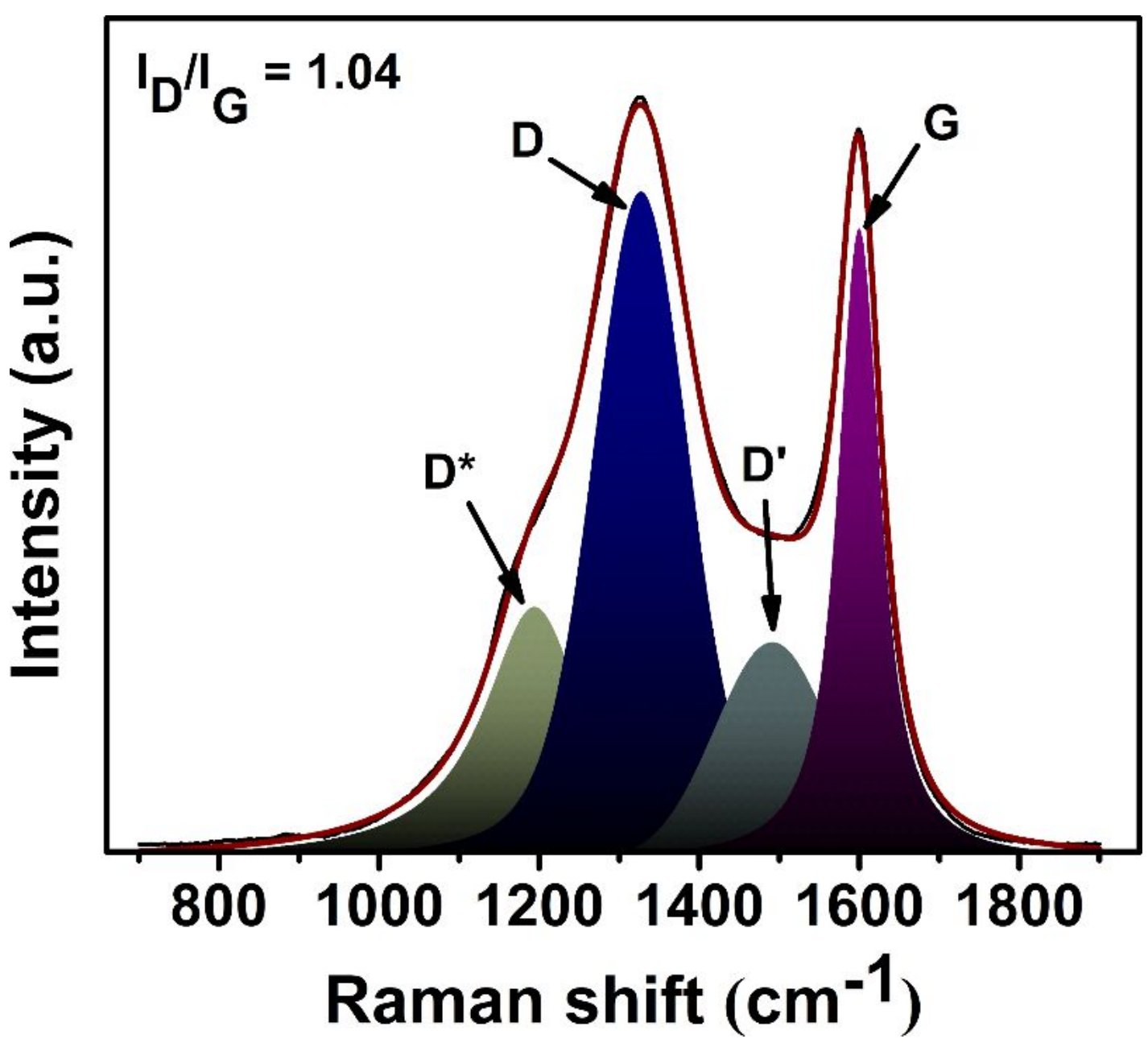

Figure S1. Deconvoluted Raman spectrum of GPB.

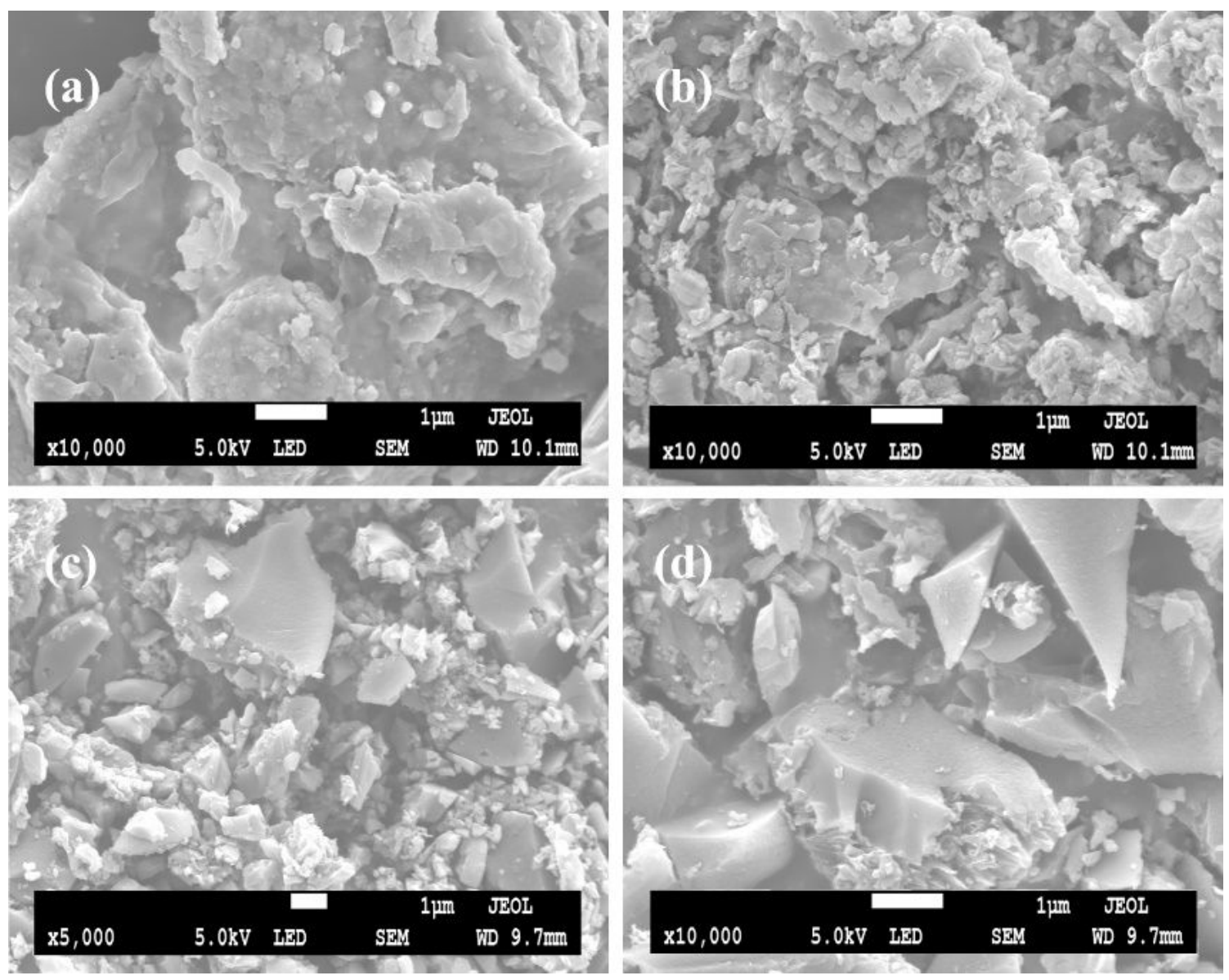

Figure S2. FESEM micrographs of (a) GP, (b) GPH, and (c and d) GPB.

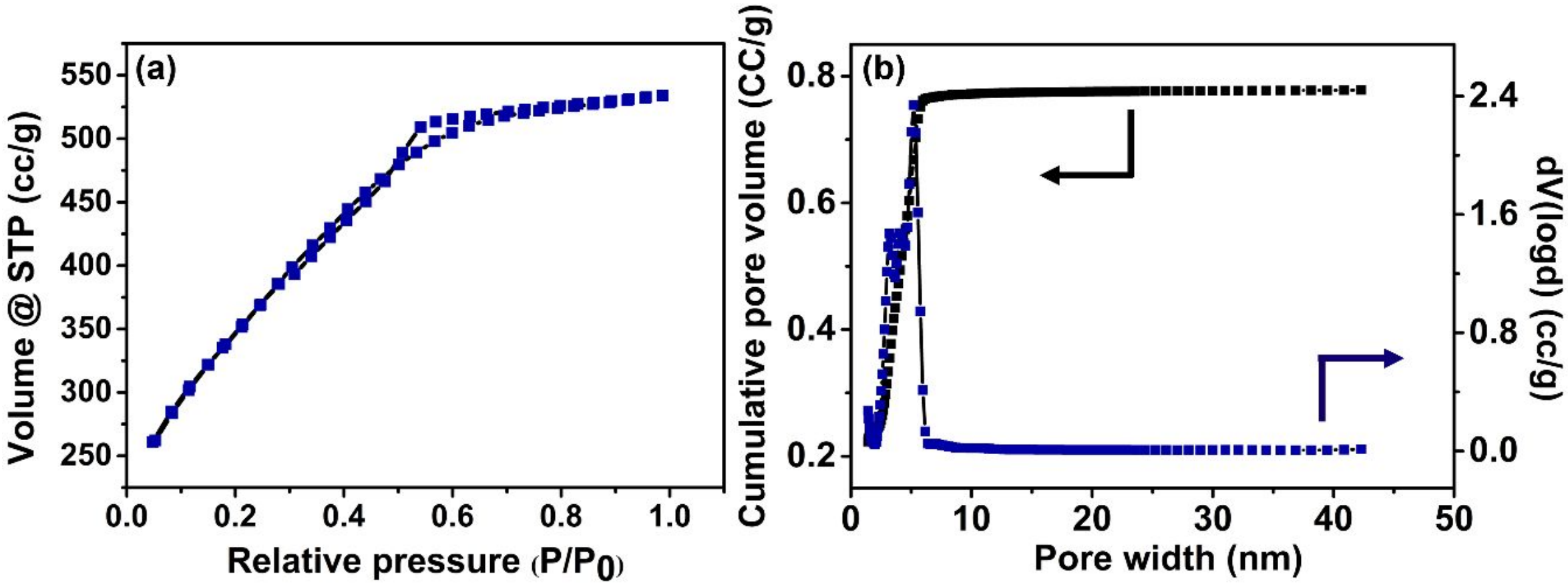

Figure S3. (a-b) N_2 adsorption/desorption isotherm, and pore diameter and pore volume distribution curves of GPB.

Dunn's kinetic analysis calculations from CV data in Figure 5 (a) have been conducted to quantitatively separate capacitive and diffusion-controlled contributions from the GPB electrochemical response. Dunn's method [1]:

$$i(V)=k_1\upsilon+k_2\upsilon^{1/2}$$

where:

$i(V)$ = measured current

υ = scan rate

$k_1\upsilon$ = capacitive contribution

$k_2\upsilon^{1/2}$ = diffusion contribution

Table S1. Capacitive and diffusion-controlled contributions to charge storage in GPB. Obtained from CV data in Figure 5 (a).

Scan rate	Capacitive (%)	Diffusion (%)
5 mV/s	60–70	30–40
20 mV/s	75–85	15–25
100 mV/s	>90	<10

Thus, the results confirm that capacitive processes dominate the overall charge storage mechanism in GPB, while diffusion-controlled contributions remain comparatively minor.

C_A has been determined with equation (S1), using data from the three-electrode configuration in KOH, i.e., Figure 5 (a) [2]:

$$C_A = \frac{C_{sp}}{SSA} \qquad \text{(S1)}$$

Thus:

$$C_A = \frac{183.5\ Fg^{-1}}{1240.7\ m^2 g^{-1}}$$

$$C_A = 0.148\ F\ m^{-2}$$

$$C_A = 14.8\ \mu F\ cm^{-2}$$

Table S2. Fitting values of the equivalent circuit elements in the three-electrodes configuration.

Electrolyte	R_s (Ω)	R_{ct} (Ω)	CPE_{dl} (F)	C_l (F)	Z_w (Ω)
		Three electrodes cell			
6 M KOH	1.27	6.19	1.975×10^{-3}	0.855	13.82
0.5 M Na_2SO_4	10.57	21.36	3.803×10^{-4}	12.83	42.33

Table S3. Fitting values of the equivalent circuit elements in the symmetric supercapacitor configuration.

Elements<br>Electrolytes	R_s<br>(Ω)	R_{ct}<br>(Ω)	R_l<br>(Ω)	CPE_{dl}<br>(F)	C_l<br>(F)	Z_w<br>(Ω)
			Two electrodes supercapacitor			
6 M KOH	0.185	0.573	$27.71 x 10^{-15}$	$74.12 x 10^{-6}$	0.126	12.31
0.5 M Na_2SO_4	1.23	16.0	$1.426 x 10^{-6}$	$0.0642 x 10^{-3}$	0.010	57.14
1 M $NaClO_4$	10.1	50.23	$1.016 x 10^{-11}$	$3.635 x 10^{-5}$	0.143	52.35

The radar chart can serve to visualize the strengths and weaknesses of 6 M KOH, 0.5 M Na_2SO_4, and 1 M $NaClO_4$ (EC|PC) in the electrochemical performance of GPB. The chart integrates multiple performance indicators into a single graphical representation.

The six axes of the radar chart represent key electrochemical metrics:

1. P_D
2. E_D
3. C_{sp}
4. Voltage window
5. R_{ct}
6. % C_R
7. Stability cycles

Data normalization:
Since each parameter has different units and magnitudes, values are normalized as:

$$X_{normal} = \frac{x_{i-}x_{min}}{x_{max}-x_{\ min}} \qquad \text{(S2)}$$

where x_i: current value of data set, x_{min}: minimum value of data set, x_{max}: maximum value of data set.

The X_{normal} values have been plotted as seen in Figure 10, allowing a direct comparison among electrolytes. A summary of the advantages of each environment is given in Table S4.

Table S4. Highlights of electrolyte performance with GPB as active material from the radar chart.

Electrolyte	Main advantage	Highlighted metric
6 M KOH	Fast OH^- transport, high ionic conductivity	C_{sp}, % C_R
0.5 M Na_2SO_4	Acceptable performance	-
1 M $NaClO_4$ (EC/PC)	High E_D, suitable for high energy storage devices	P_D, E_D, voltage window

Table S5. Comparison of GPB with garlic-based and other biomass-derived porous biocarbons in supercapacitor applications.

Active material	Electrolyte	SSA (m^2 g^{-1})	E_D (Wh kg^{-1})	P_D (W kg^{-1})	Cycling stability (%)	Ref.
Garlic peel porous carbon (GBPC-6)	6 M KOH	3887.0	59.57	190.6	97.1	[3]
Garlic peel (HGAP-7)	6 M KOH	3325.2	n.r.	n.r.	98.4	[4]
Garlic seeds (honeycomb hierarchical) h-HPC	6 M KOH	1417.0	31.7	500	99.2	[5]
Garlic peel activated carbon monolith ACM-0.50	1 M H_2SO_4	786.83	28.58	71.16	n.r.	[6]
Garlic waste nanoporous carbon (GAAC6)	3 M KOH	3449.2	31.80	8146	100	[7]
Garlic skin-derived carbon	6 M KOH	2818.0	n.r.	n.r.	94	[8]
Cassava peel carbon (CA-70)	1 M H_2SO_4	398.46	8.91	n.r.	86.34	[9]
Orange peel activated carbon	2 M Na_2SO_4	137.99	8.0147	n.r.	n.r.	[10]

GPB	6 M KOH	1240.7	3.89	98.75	91.91	This work
	0.5 M Na_2SO_4		11.57	107.0	89.02	
	1 M $NaClO_4$-(EC|PC)		19.9	183.9	78.38	

n.r.: not reported

References

[1] S. Harish, P.U. Sathyakam, Dunn's Method for Distinguishing Charge Storage Mechanisms in Supercapacitors: A Status Quo Review. J. Electron. Mater. 54, 10858–10872 (2025).

[2] C. Largeot, C. Portet, J. Chmiola, P.L. Taberna, Y. Gogotsi, P. Simon, Relation Between the Ion Size and Pore Size for an Electric Double-Layer Capacitor. J. Am. Chem. Soc. 2008, 130 (9), 2730–2731.

[3] Z. Teng, K. Han, J. Li, Y. Gao, M. Li, T. Ji, Ultrasonic-Assisted Preparation and Characterization of Hierarchical Porous Carbon Derived from Garlic Peel for High-Performance Supercapacitors. Ultrason. Sonochem. 2020, 60, 104756.

[4] T. Ji, K. Han, Z. Teng, J. Li, M. Wang, J. Zhang, et al., Synthesis of Activated Carbon Derived from Garlic Peel and Its Electrochemical Properties. Int. J. Electrochem. Sci. 2021, 16 (1), 150653.

[5] S. Li, Q. Chen, Y. Gong, H. Wang, D. Li, Y. Zhang, Q. Fu, C. Pan, "One-Step" Carbonization Activation of Garlic Seeds for Honeycomb-Like Hierarchical Porous Carbon and Its High Supercapacitor Properties. ACS Omega 2020, 5 (46), 29913–29921.

[6] E. Taer, A. Apriwandi, W.S. Mustika, M.A. Mardiah, R. Taslim, An Environmental Approach in Production of Activated Carbon Monolithic Derived from Garlic Peels for Supercapacitor Application. Trends Sci. 2023, 20 (1), 6396.

[7] J.M. Davidraj, C.I. Sathish, A.M. Ruban, V. Perumalsamy, V. Narayanan, X. Yu, et al., Functionalized Nanoporous Biocarbon Derived from Garlic Waste for Enhanced CO2 Capture and Supercapacitor Performance. Carbon 2025, 242, 120409.

[8] Q. Zhang, K. Han, S. Li, M. Li, J. Li, K. Ren, Synthesis of Garlic Skin-Derived 3D Hierarchical Porous Carbon for High-Performance Supercapacitors. Nanoscale 2018, 10 (5), 2427–2437.

[9] J. Ospino, J.P. Parra-Barraza, S. Cervera, E.E. Coral-Escobar, O.A. Vargas-Ceballos, Activated Carbon from Cassava Peel: A Promising Electrode Material for Supercapacitors. Rev. Fac. Ing., Univ. Antioquia 2022, (102), 88–95.

[10] Y.K. Lee, C.P. Liew, P.L. Kiew, B.C.Y. Foo, W.M. Yeoh, M.Y. Ho, Orange Peel-Derived Activated Carbon as a Potential Electrode Material for Supercapacitor Application. Chem. Eng. Trans. 2023, 106, 1285–1290.
